# Impacts of COVID-19 on Stress in Middle School Teachers and Staff in Minnesota: An Exploratory Study Using Random Forest Analysis

**DOI:** 10.3390/ijerph20176698

**Published:** 2023-09-01

**Authors:** Alyson B. Harding, Marizen R. Ramirez, Andrew D. Ryan, Bao Nhia Xiong, Christina E. Rosebush, Briana Woods-Jaeger

**Affiliations:** 1 Division of Environmental Health Sciences, School of Public Health, University of Minnesota, Minneapolis, MN 55455, USA; mramirez@umn.edu (M.R.R.); ryanx029@umn.edu (A.D.R.); xion1894@umn.edu (B.N.X.);; 2 Department of Environmental and Occupational Health, Program of Public Health, University of California at Irvine, Irvina, CA 92697, USA; 3 Department of Behavioral, Social and Health Education Sciences, Rollins School of Public Health, Emory University, Atlanta, GA 30322, USA; bwoodsjaeger@emory.edu

**Keywords:** COVID-19, random forest, machine learning, variable importance, occupational health, mental health, stress

## Abstract

While the COVID-19 pandemic has negatively impacted many occupations, teachers and school staff have faced unique challenges related to remote and hybrid teaching, less contact with students, and general uncertainty. This study aimed to measure the associations between specific impacts of the COVID-19 pandemic and stress levels in Minnesota educators. A total of 296 teachers and staff members from eight middle schools completed online surveys between May and July of 2020. The Epidemic Pandemic Impacts Inventory (EPII) measured the effects of the COVID-19 pandemic according to nine domains (i.e., Economic, Home Life). The Kessler-6 scale measured non-specific stress (range: 0–24), with higher scores indicating greater levels of stress. Random forest analysis determined which items of the EPII were predictive of stress. The average Kessler-6 score was 6.8, indicating moderate stress. Three EPII items explained the largest amount of variation in the Kessler-6 score: increase in mental health problems or symptoms, hard time making the transition to working from home, and increase in sleep problems or poor sleep quality. These findings indicate potential areas for intervention to reduce employee stress in the event of future disruptions to in-person teaching or other major transitions during dynamic times.

## 1. Introduction

Teaching has long been identified as a high-stress occupation. The 2017 Educator Quality of Life Survey, conducted by the American Federation of Teachers, revealed that 61% of teachers and school staff report that their job is “always” or “often” stressful, compared to 30% of the general population [1]. In the same study, educators and school staff also reported that their mental health was “not good” for an average of 7 of the last 30 days, and 21% reported their mental health as “not good” for 11 or more days in the last 30 [1]. In a 2005 study comparing work-related stress across 26 occupations in the United Kingdom, teachers ranked worse than average in physical health, psychological well-being, and job satisfaction [2]. Job turnover is high in teaching, with over 40% of new teachers in the United States leaving the profession within five years [3,4,5]. Teachers often report experiencing stress due to high job demands, including the pressure of high-stakes testing [1,4], disruptive student behavior [5,6,7], and onerous workloads [1,5]. Workplace stress is associated with negative professional outcomes, including burnout, absenteeism, and attrition [4,8]. Stress can also lead to negative health outcomes, including depression, anxiety, and suicidal ideation [8,9,10]. Additionally, studies by Herman and colleagues have found that teacher stress impacts student outcomes. Students taught by teachers with high stress and low coping skills had the lowest academic achievement scores and the highest rates of student behavioral problems compared to students taught by teachers with other profiles of stress and coping [8,11].

In addition to pre-existing stressors, teachers faced a new set of unexpected challenges with the beginning of the COVID-19 pandemic in the United States in the spring of 2020, including potential COVID-19 exposure, remote and hybrid teaching, less contact with students, and general uncertainty. Studies from around the world have begun to elucidate the toll of the COVID-19 pandemic on teachers. A review of eight studies published before June of 2021 reported that 17% of teachers were experiencing anxiety, and 19% of teachers were experiencing depression due to the pandemic [12]. A study of nearly 90,000 teachers in China conducted in the spring of 2020 found a rate of anxiety of 13.7% [13], while a survey in Morocco from the same period revealed that 54.4% of teachers were suffering from burnout [14]. Teachers are experiencing stress due to less contact with students [15,16,17] and challenges adapting to new teaching techniques [15,17,18]. Teachers and school staff also cited increased workloads [15,16,18] and struggling with work-life balance [16,17,18], as well as general uncertainty [15,16,18] as major stressors. Pandemic stress is having a major impact on the profession. In a survey of over 3500 members of the National Educators Association conducted in the United States in early 2022, 55% of respondents said they are more likely to leave or retire from education sooner than planned because of the pandemic [19].

While several studies have documented increased stress in teachers and school staff due to the pandemic, few have sought to quantify the broader impacts of the pandemic beyond work-related changes to examine how pandemic-related life shifts are associated with symptoms of stress. Just as job-related factors can contribute to overall health and well-being outside the workplace, factors beyond the workplace may exacerbate worker stress. It is important to examine a wide variety of pandemic impacts on the stress symptoms of educators. This knowledge is foundational to the development of interventions to reduce stress during future periods of great change and uncertainty.

Machine learning is a technique that is increasingly being used as an exploratory first step in understanding new phenomena and can be used to inform future hypothesis-driven research. This exploratory, cross-sectional study aimed to assess associations between self-reported impacts of the COVID-19 pandemic from 10 domains (e.g., Work and Employment, Home Life, Economic) and general stress symptoms among teachers and school staff from 8 middle schools in Minnesota. We employed random forest analysis, demonstrating the utility of the method for rapidly identifying important variables from large datasets. Understanding which pandemic factors were most predictive of stress will allow for targeted intervention development and better preparation for future disturbances to in-person teaching.

## 2. Materials and Methods

### 2.1. Study Population

The data for this study were collected through Link for Equity, an ongoing school-based trauma-informed intervention study. Link for Equity seeks to address racial trauma by building cultural humility in schools and thereby reducing teacher-to-student racial microaggressions and racial bias in discipline referrals. The study enrolled eight Minnesota middle schools from seven districts based on high suspension and violence rates and high minoritized student enrollment during the summer and fall of 2019 [20]. All teachers and school staff were eligible to participate in the study. Teachers and school staff in all eight schools completed a pre-intervention baseline survey in the fall of 2019 and a follow-up survey in the spring of 2020.

The data for this cross-sectional analysis were acquired from the spring 2020 survey, which included supplemental questions about COVID-19 stressors and a mental health symptom screener. Participants were asked to complete the survey through email invitations from school administrators or research team members. Data collection occurred via web-based surveys using Qualtrics between May and July 2020. The survey data initially contained identifying information but was de-identified prior to analysis. The University of Minnesota Institutional Review Board waived the need for written consent for these surveys.

### 2.2. Measures

#### 2.2.1. COVID-19 Impacts

The survey included the Epidemic Pandemic Impacts Inventory (EPII) to measure the specific impacts of the COVID-19 pandemic [21]. The inventory contains 92 items in 10 domains: (1) Work and Employment, (2) Education and Training, (3) Home Life, (4) Social Activities, (5) Economic, (6) Emotional Health and Well-Being, (7) Physical Health Problems, (8) Physical Distancing and Quarantine, (9) Infection History, and (10) Positive Change. For example, the Work and Employment domain includes items such as “Laid off from job or had to close own business” and “Increase in workload or work responsibilities.” The Home Life domain items include “Difficulty taking care of children in the home” and “Had to spend a lot more time taking care of a family member”. The full EPII scale is presented in Supplemental Table S1. For each item, responders can choose all that apply from: Yes (Me), Yes (Other Person in Home), No, or N/A (Not Applicable).

Grasso and colleagues developed and released the EPII in 2020 in response to the COVID-19 pandemic. At the time of analysis, they had not determined optimal scoring mechanisms or conducted validation and psychometric studies of the scale [21]. Numerous studies have used the EPII or specific questions from the scale with varying scoring procedures [22,23,24,25,26,27,28]. We dichotomized answers to the EPII to Yes (Me, Other Person in Home, or both) and No (No or N/A), as consistent with previously published studies [22,23,24,26,27,28].

#### 2.2.2. Self-Reported Non-Specific Stress

The Kessler-6, a validated scale initially developed for use in the U.S. National Health Interview Survey, measured general stress symptoms [29]. The scale contains six questions, scored on a five-point Likert scale. Questions ask, in the past month, how frequently the respondent felt: nervous, hopeless, restless or fidgety, so depressed that nothing could cheer them up, that everything was an effort, and worthless. Answers ranged from 0 (none of the time) to 4 (all of the time). The scores were summed, with total Kessler-6 scores ranging from 0 to 24, with higher scores indicating more stress symptoms. A threshold of ≥5 has been used to identify a moderate level of mental distress that impacts functioning and necessitates treatment [30]. A cut-point of ≥13 has been used to determine serious mental illness, defined as meeting the diagnostic criteria for a DSM-IV disorder and experiencing impairment [29]. We used the total Kessler-6 score as a continuous measure for the model outcome.

### 2.3. Random Forests

We used random forests to identify which EPII items were most strongly associated with the total Kessler-6 score, a measure of non-specific stress symptoms. Random forests are often used to identify predictive variables, ranked in terms of “variable importance”. Random forests have been used to establish variable importance in public health research across a wide variety of topics, including predicting violence [31,32], assessing biosecurity practices [33], and examining phenotypic risk factors for temporomandibular disorders [34]. Random forests are useful for rapidly assessing large, complex datasets, and this method allowed us to examine all 92 items of the EPII without a priori assumptions. Random forests are an ensemble machine learning method that combines multiple iterations of decision trees, known as classification or regression trees. Decision trees use recursive binary splitting to partition data [35]. Data are split into two groups based on the variable that most reduces the variance of the outcome. Within each group, the data are further partitioned based on the variable that most reduces the variance of the outcome in that group. Data are further partitioned until data are split into terminal nodes when the data cannot be split further to improve model fit.

Decision trees are beneficial for handling high-dimensional data and complex, non-linear interactions between variables [35]. They do not require normal distributions of numeric data and are robust to outliers. However, decision trees have high variance and can be prone to overfitting [35]. To overcome this issue, random forests aggregate multiple decision trees. Each tree is created using a bootstrapped random subset of observations and a random sample of predictors, resulting in less correlation between trees compared to trees that include all possible predictors [35,36]. Averaging these independent, uncorrelated trees leads to a better reduction in variance than if the trees were highly correlated.

We chose to use random forest for this exploratory analysis, as we were interested in identifying important variables rather than attempting to construct a full predictive or causal model. This is a key first step in exploring a new phenomenon, specifically pandemic stress in teachers.

### 2.4. Statistical Analysis

We built a random forest model using all 92 items from all subscales of the Epidemic Pandemic Impacts Inventory (EPII) as predictors and the Kessler-6 score as the outcome to establish variable importance related to general stress. We optimized the number of variables selected for each tree and the minimum number of data points required in a node to split that node further based on reducing the mean absolute error of the final model. We used 500 trees and employed 5-fold cross-validation. Variables were ranked for importance by percent increase in mean squared error (MSE) when the variable was permuted [37]. Analyses were performed using R statistical software (v.4.0.3) [38] using packages within tidymodels [39] and the randomforest package [37].

### 2.5. Missing Data

Before analysis, we removed observations that were missing responses to more than 75% of the EPII inventory items. We also removed observations missing the outcome variable, the total Kessler-6 score. We did not impute these variables, as they were the primary exposure and outcome of interest. After removing those participants, only 0.5% of the data was missing. All missing predictor values were set to zero (“no”). We conducted sensitivity analyses with missing predictor values set to one (“yes”) and with complete cases (removed observations with missing predictive values). The results did not vary substantially among the three methods for missing data.

## 3. Results

### 3.1. Population

A total of 366 teachers and school staff members from the eight middle schools provided data via the Qualtrics survey between May and July 2020. Two hundred and ninety-six participants completed at least 75% of the EPII scale and completed the Kessler-6 scale and were included in this analysis. Respondents identified predominately as white, non-Hispanic, and female (Table 1). Approximately three-quarters of respondents were teachers, while the remaining respondents were administrators, support staff, or other roles. Participants excluded from the study due to missing data were more likely to be female, to have worked in education for under ten years, and were younger, on average, than study participants.

### 3.2. Measures

Stress symptoms, measured by the Kessler-6 scale, ranged from 0 to the maximum possible value of 24. The median and mean Kessler-6 scores were 6.0 and 6.8, respectively, indicating moderate mental distress. Two-thirds of participants in this study population had Kessler-6 scores greater than or equal to five, the threshold for indicating moderate levels of mental distress. Severe mental illness, defined as a score of 13 or higher, was detected in 11.5% of this study population. Stress levels did not differ between respondents who completed the survey before the estimated summer school break (June 15) and those who completed it after the break. Additionally, stress levels did not differ between respondents from schools receiving the Link for Equity intervention and respondents from control schools.

The number of pandemic impacts experienced by participants varied substantially, ranging from 9 to 54 items of the EPII, with an average of more than 26 impact items (Table 2). Participants reported experiencing a high number of impacts from the Social Activities domain, with more than 80% of respondents reporting being separated from family or close friends, having family celebrations canceled or restricted, having planned travel or vacations canceled, and being unable to perform enjoyable activities or hobbies (see Table in Table S1). Participants reported a high number of pandemic impacts from the Work and Employment and Positive Changes domains. A few study participants reported impacts from the Infection History, Physical Distancing and Quarantine, and Economic domains.

### 3.3. Random Forest Model

**Figure 1:**
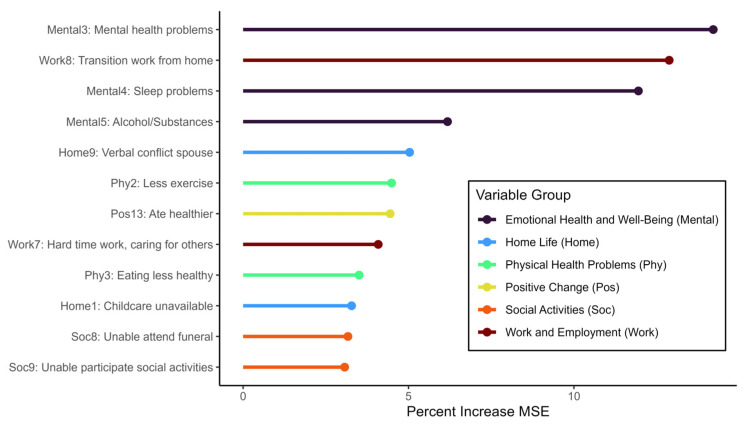
Variable Importance Plot. The variable importance plot identifies which items from the Epidemic Pandemic Impacts Inventory (EPII) are most predictive of the Kessler-6 score, or general stress, in our population. We identified variable importance based on the percent increase in mean squared error (MSE) of the final model when the variable was permuted.

Three impacts of the COVID-19 pandemic explained the largest amount of variation in the Kessler-6 score: “increase in mental health problems or symptoms,” “hard time making the transition to working from home,” and “increase in sleep problems or poor sleep quality” (Figure 1). That is, when these variables were permuted, the respective increases in mean squared error for the final model were greater than for all other variables tested.

## 4. Discussion

Pandemic stress is a new phenomenon that will likely continue, and this study provides a unique opportunity to begin to explore factors related to stress through machine learning. In this exploratory analysis, we sought to identify which items of the EPII explained the most variability in the Kessler-6 score, a measure of non-specific stress. Random forests are a useful tool for establishing variable importance and have been used in a wide variety of different contexts [31,32,33,34]. Unlike traditional regression techniques, which use indirect metrics such as *p*-values and measures of model fit to establish variable importance, random forests compute internal metrics for variable importance by calculating the change in model mean squared error when each variable is randomly permuted [31,36,37]. Additionally, random forests are useful for high-dimensional data with complex interactions and do not rely on the assumption of linearity. In this case, we were able to assess all 92 EPII items without making a priori assumptions.

Our study reflected that teachers and school staff were experiencing stress in the spring of 2020, with two-thirds of survey respondents reporting moderate stress levels. This is consistent with other studies that reported challenges among teachers and school staff due to the COVID-19 pandemic and increased stress as a result [12,15,16,17,19]. Before the COVID-19 pandemic, it was estimated that 6% of adults in the United States met the criteria of severe mental illness [30]; however, severe mental illness was detected in 11.5% of this study population.

Of the top three impacts of COVID-19 identified as predictors of stress levels, two were from the Emotional Health and Well-Being subscale. The fact that the items “increase in mental health problems” and “increase in sleep problems or poor sleep quality” were predictors of stress is not surprising. The item “increase in mental health problems” likely measured a similar construct as the Kessler-6 scale. The identification of this item as a top predictor of non-specific stress symptoms reinforces the validity of using random forests to establish top EPII items associated with stress.

The literature has established the relationship between sleep and stress across multiple populations [40,41,42,43]. Åkerstedt and colleagues found bedtime stress and worry to be the main predictor of sleep quality [44]. Conversely, a 2021 meta-analysis of randomized control trials found that improving sleep led to better quality mental health [45]. Additional studies have found high levels of stress and decreased sleep quality during the COVID-19 pandemic [46,47]. These findings suggest that teachers and school staff may benefit from mental health and well-being resources or interventions. Numerous web- and app-based interventions have demonstrated positive impacts on employee mental health [48,49], including interventions that focused specifically on improving sleep quality [50,51].

The top predictor outside the Emotional Health and Well-Being scale was “hard time adjusting to working from home” from the “Work and Employment” domain. Previous findings have identified challenges adapting to new teaching techniques and struggling with work-life balance as stressors for teachers during COVID-19 [15,16,17,18]. This presents an important potential intervention. The COVID-19 pandemic was an unexpected disruption to life, forcing teachers and school staff in Minnesota and around the world to quickly adapt to working remotely. Unfortunately, such disruptions may occur again in the future, from disease or disasters such as hurricanes, flooding, or wildfires. Having a plan to ease the transition to working from home and accommodating teachers and school staff may reduce stress if the situation arises in the future.

Other studies have used portions of the EPII to examine associations between COVID-19 impacts and mental health outcomes in various populations. Alzueta and colleagues surveyed over 6800 individuals from 59 countries during the spring of 2020, asking 21 of the 92 EPII items. Their study concluded that COVID-19-related life changes explained a higher proportion of the variance of depression and anxiety compared to demographics, COVID-19 exposure, or quarantine level [22]. A sub-analysis of the Alzueta study focusing on older adults identified having a hard time working from home and being separated from family and close friends as predictors of both depression and anxiety [27]. The study identified an increase in arguments with other adults in the home as the largest predictor of anxiety. Although our study examined non-specific stress, we also identified having a hard time working from home as an important pandemic impact. However, being separated from family and close friends and increases in arguments with other adults in the home were not identified as top predictors of stress in our population, likely due to demographic and cultural differences. These studies further highlight the importance of examining the specific impacts of the COVID-19 pandemic and the utility of the EPII.

This study had limitations. Due to the study’s cross-sectional nature, it is not possible to determine temporality, so there is no evidence of a direction between the impacts of COVID-19 and stress levels. We surveyed teachers and staff at eight middle schools across Minnesota in urban, suburban, and rural areas. Additionally, these schools were already enrolled in the Link for Equity study due to their high rates of suspension and school violence. Results may not be generalizable to other schools, particularly those with vastly different policies related to school closures during COVID-19 and those with different baseline characteristics and available resources. Study findings were limited to teachers and school staff employed in Minnesota schools that had a large enrollment of Black, Latinx, and American Indian students. Future research could employ a similar study design to determine if results differed between occupational groups or geographic locations.

Despite these limitations, we demonstrated the utility of using random forest analysis to establish variable importance, exploring the association of the Epidemic Pandemic Impacts Inventory (EPII) items and stress symptoms among middle school teachers and staff members in Minnesota. Since the EPII currently lacks established scoring mechanisms, our study offers a possible mechanism for evaluating variable importance among EPII items. Random forests can be used to identify important predictors of an outcome from large datasets, including those derived from electronic medical records, census data, genetic data, or survey data, as we demonstrated here. Random forests, a machine learning technique, is also shown to be an appropriate technique for initial rapid exploration of a new phenomenon and can yield results that indicate areas for future study.

## 5. Conclusions

Variable importance from random forest analysis identified “increase in mental health problems” and “increase in sleep problems or poor sleep quality” as top predictors of stress in a population of Minnesota middle school teachers and staff at the start of the COVID-19 pandemic. These findings suggest that teachers and school staff may benefit from additional mental health and well-being resources or interventions, especially those focused on improved sleep quality. The other top predictor of stress, “hard time making the transition to working from home,” indicates that employers should create plans and establish protocols to ease their employees’ transition to working from home or other necessary transitions as we continue to live in dynamic times. This study also demonstrated the utility of using random forest analysis to identify important variables from large datasets. Future environmental and public health studies involving large, complex datasets may consider random forests or other machine-learning methods of analysis.

## Figures and Tables

**Table 1:** Participant characteristics.

Characteristic	n (%)
Gender	
Male	58 (19.6%)
Female	185 (62.5%)
Missing	53 (17.9%)
Age	
<30	34 (11.4%)
30–39	88 (29.7%)
40–49	87 (29.4%)
50–59	57 (19.3%)
60+	23 (7.8%)
Missing	7 (2.4%)
Hispanic	
Yes	4 (1.4%)
No	240 (81.1%)
Missing	52 (17.6%)
Race	
American Indian or Alaska Native	5 (1.7%)
Asian	4 (1.4%)
Black or African American	7 (2.3%)
White	217 (73.3%)
Other	2 (0.7%)
Multiple races	6 (2.0%)
Missing	55 (18.6%)
Role	
Administrator	6 (2.0%)
Teacher	225 (76.0%)
Support Staff/Other	63 (21.3%)
Missing	2 (0.7%)
Years working in education	
0–3 years	29 (9.8%)
4–9 years	54 (18.2%)
10+ years	163 (55.1%)
Missing	50 (16.9%)

**Table 2:** Summary of Epidemic Pandemic Impacts Inventory responses, by domain and overall.

Domain	Number of Questions	Reported Range	Mean	Median
Work and Employment	11	0–10	5.40	5
Education and Training	2	0–2	0.63	1
Home Life	13	0–8	1.76	1
Social Activities	10	0–10	5.83	6
Economic	5	0–4	0.14	0
Emotional Health and Well-Being	8	0–8	2.92	3
Physical Health Problems	8	0–7	2.84	3
Physical Distancing and Quarantine	8	0–7	1.30	1
Infection History	8	0–3	0.19	0
Positive Change	19	0–18	8.78	9
Total EPII scale	92	9–54	26.69	26

## Data Availability

De-identified data will be made available through the Data Repository for the University of Minnesota (DRUM) at the conclusion of this study.

## Supplementary Materials

The following supporting information can be downloaded at: https://www.mdpi.com/article/10.3390/ijerph20176698/s1, Table S1: Epidemic Pandemic Impacts Inventory (EPII) Responses.
