# Supplementary material for: Impacts of COVID-19 on Stress in Middle School Teachers and Staff in Minnesota: An Exploratory Study Using Random Forest Analysis
Source: Int J Environ Res Public Health. 2023 Sep 1;20(17):6698. doi: 10.3390/ijerph20176698 (PMC10487626; doi:10.3390/ijerph20176698)
Supplement: Supplementary file 1 [file ijerph-20-06698-s001.zip › ijerph-2503582-supplementary.pdf]

Table S1: Epidemic Pandemic Impacts Inventory (EPII) Responses

EPII Item	Yes (%)*	No (%)
**Work and Employment**		
Work1: Laid off from job or had to close own business.	39 (13.2%)	256 (86.8%)
Work2: Reduce work hours or furloughed.	80 (27.0%)	216 (73.0%)
Work3: Had to lay-off or furlough employees or people supervised.	15 (5.1%)	280 (94.9%)
Work4: Had to continue to work even though in close contact with people who might be infected (e.g., customers, patients, co-workers).	107 (36.4%)	187 (63.6%)
Work5: Spend a lot of time disinfecting at home due to close contact with people who might be infected at work.	97 (32.9%)	198 (67.1%)
Work6: Increased in workload or work responsibilities.	157 (53.4%)	137 (46.6%)
Work7: Hard time doing job well because of needing to take care of people in the home.	102 (34.6%)	193 (65.4%)
Work8: Hard time making transition to working from home.	161 (55.3%)	130 (44.7%)
Work9: Provided direct care to people with the disease (e.g., doctor, nurse, patient case assistant, radiologist).	11 (3.7%)	284 (96.3%)
Work10: Provided supportive care to people with the disease (e.g., medical support staff, custodial, administration).	17 (5.8%)	277 (94.2%)
Work11: Provided care to people who died as a result of the disease.	6 (2.0%)	287 (98.0%)
**Education and Training**		
Edu1: Had a child in home who could not go to school.	147 (49.8%)	148 (50.2%)
Edu2: Adult unable to go to school or training for weeks or had to withdraw.	38 (13.2%)	249 (86.8%)
**Home Life**		
Home1: Childcare or babysitting unavailable when needed.	54 (18.2%)	242 (81.8%)
Home2: Difficulty taking care of children in the home.	68 (23.1%)	227 (76.9%)
Home3: More conflict with child or harsher in discipling child or children.	58 (19.6%)	238 (80.4%)
Home4: Had to take over teaching or instructing child.	117 (39.8%)	177 (60.2%)
Home5: Family or friends had to move into your home.	20 (6.8%)	275 (93.2%)
Home6: Had to spend a lot more time taking care of a family member.	84 (28.5%)	211 (71.5%)
Home7: Had to move or relocate.	12 (4.1%)	284 (95.9%)
Home8: Became homeless.	1 (0.3%)	295 (99.7%)
Home9: Increase in verbal arguments or conflict with a partner or spouse.	70 (23.6%)	226 (76.4%)
Home10: Increase in physical conflict with a partner or spouse.	1 (0.3%)	295 (99.7%)
Home11: Increase in verbal arguments or conflict with other adult(s) in home.	22 (7.4%)	274 (92.6%)
Home12: Increase in physical conflict with other adult(s) in home.	2 (0.7%)	292 (99.3%)
Home13: Increase in physical conflict among children in home.	10 (3.4%)	286 (96.6%)
**Social Activities**		
Soc1: Separated from family or close friends.	254 (86.1%)	41 (13.9%)
Soc2: Did not have the ability or resources to talk to family or friends while separated.	20 (6.8%)	275 (93.2%)

Soc3: Unable to visit loved one in a care facility (e.g., nursing home, group home).	97 (32.8%)	199 (67.2%)
Soc4: Family celebrations cancelled or restricted.	278 (93.9%)	18 (6.1%)
Soc5: Planned travel or vacations cancelled.	247 (83.7%)	48 (16.3%)
Soc6: Religious or spiritual activities cancelled or restricted.	194 (65.8%)	101 (34.2%)
Soc7: Unable to be with a close family member in critical condition.	65 (22.0%)	230 (78.0%)
Soc8: Unable to attend in-person funeral or religious services for a family member or friend who died.	90 (30.5%)	205 (69.5%)
Soc9: Unable to participate in social clubs, sports teams, or usual volunteer activities.	235 (79.4%)	61 (20.6%)
Soc10: Unable to do enjoyable activities or hobbies.	238 (81.2%)	55 (18.8%)
**Economic**		
Economic1: Unable to get enough food or healthy food.	7 (2.4%)	288 (97.6%)
Economic2: Unable to access clean water.	1 (0.3%)	294 (99.7%)
Economic3: Unable to pay important bills like rent or utilities.	8 (2.7%)	287 (97.3%)
Economic4: Difficult getting places due to less access to public transportation or concerns about safety.	15 (5.1%)	281 (94.9%)
Economic5: Unable to get needed medications (e.g., prescriptions or over-the-counter).	11 (3.7%)	284 (96.3%)
**Emotional Health and Well-Being**		
Mental1: Increase in child behavioral or emotional problems	81 (27.4%)	215 (72.6%)
Mental2: Increase in child's sleep difficulties or nightmares.	58 (19.6%)	238 (80.7%)
Mental3: Increase in mental health problems or symptoms (e.g., mood, anxiety, stress)	172 (58.3%)	123 (41.7%)
Mental4: Increase in sleep problems or poor sleep quality.	164 (55.4%)	132 (44.6%)
Mental5: Increase in use of alcohol or substances.	84 (28.4%)	212 (71.6%)
Mental6: Unable to access mental health treatment or therapy.	24 (8.1%)	272 (91.9%)
Mental7: Not satisfied with changes in mental health treatment or therapy.	11 (3.7%)	283 (96.3%)
Mental8: Spent more time on screens and devices (e.g., looking at phone, playing video games, watching TV).	268 (90.5%)	28 (9.5%)
**Physical Health Problems**		
Phy1: Increase in health problems not related to this disease.	43 (14.5%)	253 (85.5%)
Phy2: Less physical activity or exercise.	179 (60.5%)	117 (39.5%)
Phy3: Overeating or eating more unhealth foods (e.g., junk food).	181 (61.6%)	113 (38.4%)
Phy4: More time sitting down or being sedentary.	261 (88.5%)	34 (11.5%)
Phy5: Important medical procedure cancelled (e.g., surgery).	30 (10.2%)	265 (89.2%)
Phy6: Unable to access medical care for a serious condition (e.g., dialysis, chemotherapy).	5 (1.7%)	290 (98.3%)
Phy7: Got less medical care than usual (e.g., routine or preventative care appointments).	127 (42.9%)	169 (57.1%)
Phy8: Elderly or disabled family member not in home unable to get the help they need.	19 (6.5%)	274 (93.5%)
**Physical Distancing and Quarantine**		
Dist1: Isolated or quarantined due to possible exposure to this disease.	83 (28.0%)	213 (72.0%)

Dist2: Isolated or quarantined due to symptoms of this disease.	19 (6.4%)	276 (93.6%)
Dist3: Isolated due to existing health conditions that increase risk of infection or disease.	81 (27.6%)	213 (72.4%)
Dist4: Limited physical closeness with child or loved one due to concerns of infection.	102 (34.5%)	194 (65.6%)
Dist5: Moved out or lived away from family due to a high-risk job (e.g., health care worker, first responder).	3 (1.0%)	293 (99.0%)
Dist6: Close family member not in the home was quarantined.	42 (14.2%)	254 (85.8%)
Dist7: Family member was unable to return home due to quarantine or travel restrictions.	18 (6.1%)	277 (93.9%)
Dist8: Entire household was quarantined for a week or longer.	37 (12.5%)	258 (87.5%)
**Infection History**		
Inf1: Currently have symptoms of this disease but have not been tested.	2 (0.7%)	294 (99.3%)
Inf2: Tested and currently have this disease.	2 (0.7%)	294 (99.3%)
Inf3: Had symptoms of this disease but never tested.	31 (10.5%)	265 (89.5%)
Inf4: Tested positive for this disease but no longer have it.	1 (0.3%)	295 (99.7%)
Inf5: Got medical treatment due to severe symptoms of this disease.	1 (0.3%)	295 (99.7%)
Inf6: Hospital stay due to this disease.	0	296 (100%)
Inf7: Someone died of this disease while in our home.	0	295 (100%)
Inf8: Death of a close friend or family member from this disease.	18 (6.1%)	278 (93.9%)
**Positive Change**		
Pos1: More quality time with family or friends in person or from a distance (e.g., on the phone, Email, social media, video conferencing, online gaming).	236 (79.7%)	60 (20.3%)
Pos2: More quality time with partner or spouse.	205 (70.7%)	85 (29.3%)
Pos3: More quality time with children.	191 (67.5%)	92 (32.5%)
Pos4: Improved relationship with family or friends.	154 (52.7%)	138 (47.3%)
Pos5: New connections made with supportive people.	90 (30.5%)	205 (69.5%)
Pos6: Increase in exercise or physical activity.	117 (40.1%)	175 (59.9%)
Pos7: More time in nature or being outdoors.	229 (77.4%)	67 (22.6%)
Pos8: More time doing enjoyable activities (e.g., reading books, puzzles).	208 (70.7%)	86 (29.3%)
Pos9: Developed new hobbies or activities.	119 (40.5%)	175 (59.5%)
Pos10: More appreciative of things usually taken for granted.	249 (84.1%)	47 (15.9%)
Pos11: Paid more attention to personal health.	166 (56.1%)	130 (43.9%)
Pos12: Paid more attention to preventing physical injuries.	92 (31.3%)	202 (68.7%)
Pos13: Ate healthier foods.	116 (39.5%)	178 (60.5%)
Pos14: Less use of alcohol or substances.	38 (13.3%)	247 (86.7%)
Pos15: Spent less time on screens or devices outside of work hours (e.g., looking at phone, playing video games, watching TV).	26 (8.9%)	267 (91.1%)
Pos16: Volunteered time to help people in need.	70 (24.0%)	222 (76.0%)
Pos17: Donated time or goods to cause related to this disease (e.g., made masks, donated blood, volunteered).	110 (37.3%)	185 (62.7%)
Pos18: Found greater meaning in work, employment, or school.	117 (39.5%)	179 (60.5%)
Pos19: More efficient or productive in work, employment, or school.	105 (36.0%)	187 (64.0%)

*Numbers may not sum to 296 due to missing data
